# Draft Genome Sequence of “*Terrisporobacter othiniensis*” Isolated from a Blood Culture from a Human Patient

**DOI:** 10.1128/genomeA.00042-15

**Published:** 2015-03-05

**Authors:** Lars Christian Lund, Thomas Vognbjerg Sydenham, Silje Vermedal Høgh, Marianne Skov, Michael Kemp, Ulrik Stenz Justesen

**Affiliations:** Department of Clinical Microbiology, Odense University Hospital, Odense, Denmark

## Abstract

“Terrisporobacter othiniensis” (proposed species) was isolated from a blood culture. Genomic DNA was sequenced using a MiSeq benchtop sequencer (Illumina) and assembled using the SPAdes genome assembler. This resulted in a draft genome sequence comprising 3,980,019 bp in 167 contigs containing 3,449 coding sequences, 7 rRNAs, and 58 tRNAs.

## GENOME ANNOUNCEMENT

Reclassification of the species Clostridium glycolicus and Clostridium mayombei led to the creation of the new genus *Terrisporobacter* (1). Here, we present the addition of a possible novel species to this genus, referred to as *othiniensis* (Latin for the city Odense, where it was first isolated). “Terrisporobacter othiniensis” (proposed species) was isolated from a human blood culture as the causative agent in a case of sepsis. It is therefore assumed that this species might have pathogenic potential. It is a Gram-positive, rod-shaped, anaerobic bacterium which could not be identified with classical phenotypic methods or the Biotyper (Bruker) or Vitek M.S. (bioMérieux) MALDI-TOF mass spectrometry platforms. Partial 16S rRNA gene sequencing (452 bp) revealed its closest relative to be Terrisporobacter mayombei (98.67% identity with the type strain DSM 6539), which was isolated from the gut of the African soil-feeding termite Cubitermes speciosus (2), as a part of the termite’s microbiota. The other known member of the *Terrisporobacter* genus, T. glycolicus, is a soil bacterium (3).

The genomic DNA of “T. othiniensis” was purified according to the protocol using the DNeasy blood and tissue (Qiagen). Paired-end libraries with a calculated average insert size of 350 bp were generated using the Nextera DNA sample preparation kit (Illumina) according to the manufacturer’s protocol. DNA was sequenced on a MiSeq benchtop sequencer (Illumina) with 150 bp reads and a theoretical coverage of 30×. Any adapter contamination was removed using the sequencer’s built-in read trimming tool. Overlapping reads were merged into long reads using PEAR version 0.9.5 (4) and assembled using SPAdes version 3.1.1 (5) with the “--careful” option and the default *k* values.

The final assembly consisted of 167 contigs comprising 3,980,019 bp with an *N*_50_ of 55,536 bp and a GC content of 28.53%. Annotation was carried out using two tools: the RAST server (6) and NCBI’s PGAP annotation pipeline (7). RAST annotation resulted in 3,892 coding sequences, while PGAP identified 3,449 coding sequences and 58 tRNAs. rRNA sequences were predicted using the RNAmmer server (8). One 16S, one 23S, and eight 5S rRNA encoding genes were identified. None of the 59 clostridial virulence genes supported by experimental evidence, listed in the PATRIC (9) database, were present in the genome.

### Nucleotide sequence accession numbers.

This whole-genome shotgun project has been deposited in DDBJ/ENA/GenBank under the accession number JWHR00000000. The version described in this paper is the first version, JWHR01000000.
